# Structures of Silver Fingers and a Pathway to Their Genotoxicity

**DOI:** 10.1002/anie.202116621

**Published:** 2022-02-03

**Authors:** Katarzyna Kluska, Giulia Veronesi, Aurélien Deniaud, Bálint Hajdu, Béla Gyurcsik, Wojciech Bal, Artur Krężel

**Affiliations:** ^1^ Department of Chemical Biology Faculty of Biotechnology University of Wrocław F. Joliot-Curie 14a 50-383 Wrocław Poland; ^2^ Univ. Grenoble Alpes CNRS CEA IRIG Laboratoire de Chimie et Biologie des Métaux 38000 Grenoble France; ^3^ Department of Inorganic and Analytical Chemistry Faculty of Science and Informatics University of Szeged Dóm tér 7 6720 Szeged Hungary; ^4^ Institute of Biochemistry and Biophysics Polish Academy of Sciences Pawińskiego 5a 02-106 Warsaw Poland

**Keywords:** DNA Binding, Silver Clusters, Zinc Fingers, X-ray Absorption Spectroscopy

## Abstract

Recently, we demonstrated that Ag^I^ can directly replace Zn^II^ in zinc fingers (ZFs). The cooperative binding of Ag^I^ to ZFs leads to a thermodynamically irreversible formation of silver clusters destroying the native ZF structure. Thus, a reported loss of biological function of ZF proteins is a likely consequence of such replacement. Here, we report an X‐ray absorption spectroscopy (XAS) study of Ag_*n*_S_*n*_ clusters formed in ZFs to probe their structural features. Selective probing of the local environment around Ag^I^ by XAS showed the predominance of digonal Ag^I^ coordination to two sulfur donors, coordinated with an average Ag−S distance at 2.41 Å. No Ag−N bonds were present. A mixed AgS_2_/AgS_3_ geometry was found solely in the CCCH Ag^I^−ZF. We also show that cooperative replacement of Zn^II^ ions with the studied Ag_2_S_2_ clusters occurred in a three‐ZF transcription factor protein 1MEY#, leading to a dissociation of 1MEY# from the complex with its cognate DNA.

## Graphical abstract

The cooperative Ag^I^ binding to CCHH zinc fingers causes zinc ion release followed by a collapse of the stable secondary structure and formation of a highly stable Ag_2_S_2_ cluster. CCHC, CCCH and CCCC zinc fingers react analogously. Such silver‐induced perturbation inhibits binding of the CCHH zinc finger protein to its cognate DNA sequence and as a consequence may affect many cellular processes including transcriptional regulation.

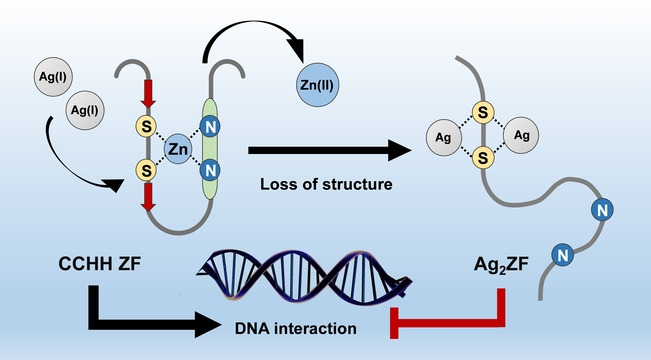

## Introduction

**Figure 1:**
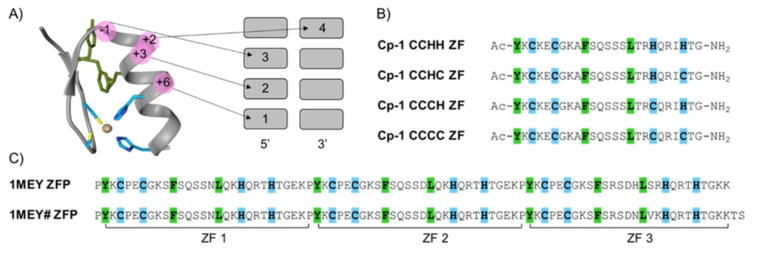
Structure and sequences of ZFs used in this study. A) 3D CCHH ZF fold adopted after Zn^II^ binding derived from a crystal structure of the 1^st^ ZF from designed Cp‐1‐like ZF protein (PDB: 1MEY). The residues shown in blue and green are key residues that bind to Zn^II^ and form a hydrophobic core, respectively. Sequence‐specific DNA‐binding mode of the CCHH ZF is shown, and the key amino acids involved in interactions with DNA are highlighted in pink. B) Sequences of the Cp‐1 ZF (2015) variants investigated in this study. The metal binding amino acids and hydrophobic residues are highlighted in blue and green, respectively. C) The amino acid sequence of the ZF protein 1MEY ZFP aligned with a sequence of the ZFP used in this study 1MEY# ZFP. Zn^II^ binding residues are highlighted in blue and hydrophobic residues are highlighted in green.

Zinc finger (ZF) proteins play important roles in interactions with nucleic acids, other proteins, and even lipids to facilitate numerous biological processes.^[1]^ The CCHH ZF motif is the most common one.[^2^, ^3^, ^4^] It is characterized by Zn^II^ binding via highly conserved cysteinyl (Cys) and histidinyl (His) couples to stabilize the protein fold, further enhanced by conserved hydrophobic residues, which form a hydrophobic core during the metal‐coupled folding process. The resulting characteristic ββα fold guarantees spatial orientation of dedicated amino acid residues, enabling specific contacts with DNA sequences or structures (Figure 1A). The database of 131 CCHH ZF sequences available in 1991 encouraged J. Berg et al. to define the consensus ZF (consensus peptide 1, Cp‐1) by aligning these sequences and picking the most populated amino acid residues for each position.^[5]^ The Cp‐1 sequence has been widely used as a model ZF in studies aimed at the understanding of kinetics and thermodynamics of metal‐coupled folding mechanisms, however CCHC, CCCH and CCCC ZF were also discovered.[^6^, ^7^, ^8^] Also, the data accumulation over the next 25 years empowered by new technologies produced 13 456 distinct CCHH ZF sequences by 2016, leading to the Cp‐1 sequence redefinition.^[9]^

Many studies demonstrated that heavy metals may substitute Zn^II^ in their native ZF coordination environment.[^1^, ^10^, ^11^, ^12^, ^13^, ^14^, ^15^] Such displacement induces conformational changes and alteration or loss of function. Ag^I^ ions have been vigorously tested for antimicrobial, anti‐inflammatory, or anticancer properties,[^16^, ^17^, ^18^, ^19^, ^20^] but their interactions with ZFs have been barely studied.^[21]^ The use of elemental silver nanoparticles (AgNPs) is rising rapidly, and nowadays, 30 % of NP products are declared to contain AgNPs. Not only medical devices or bone implants but even daily use products, including cosmetics, bedding, sportswear, protective gear (such as masks heavily used during COVID‐19 pandemics), and food containers are coated with AgNPs for their antibacterial properties.[^22^, ^23^] Rapid expansion of these coatings elevates the population exposure to AgNPs and Ag^I^ ions. Recently, high accumulation of silver in the liver and inhibition of hepatocyte nuclear receptors (NR) activity have been reported.[^24^, ^25^, ^26^] The silver reactivity with biomolecules is enabled by its high tendency to bind soft Lewis bases such as thiols, posing a question whether ZFs may be targets for Ag^I^ toxicity, e.g. by Zn^II^ displacement. If so, what are biological consequences of such toxicity? Recently, we published a study of Ag^I^ binding to the redefined Cp‐1 CCHH ZF peptide and its sequence variations (CCCH and CCCC ZFs).^[27]^ Using UV/Vis and CD spectroscopies, molecular dynamics calculations, and mass spectrometry (IM–MS), we demonstrated that Ag^I^ addition to these peptides resulted in Zn^II^ dissociation and the ββα fold collapse. Various Ag^I^−ZF complexes were detected, having the maximum number of bound Ag^I^ ions equal to the number of Cys residues in the ZF core (Ag_n_S_n_ sites), Ag_2_L, Ag_3_L, and Ag_4_L for Cp‐1 CCHH, CCCH, and CCCC ZFs, respectively. The Ag^I^ affinity for the respective complexes was at least hundred‐fold higher than that of Zn^II^.^[27]^ These data lacked detailed structural information, however, and as far as we know, no other structural studies on Ag^I^−ZF complexes have been reported. Herein, we have probed the Ag^I^ coordination environment in Cp‐1 ZFs (Figure 1B) using X‐ray absorption spectroscopy (XAS). XAS can selectively interrogate a chosen metal in a metalloprotein or metal complex, probe its average coordination sphere, and provide its bond lengths with high‐resolution (≈0.01 Å). The combined analysis of the near‐edge (XANES, X‐ray Absorption Near Edge Structure) and extended (EXAFS, Extended X‐ray Absorption Fine Structure) regions of the X‐ray absorption spectra provide the metal binding geometry, the nature of the ligands and the relative bond lengths, as well as the thermal and structural disorder.[^28^, ^29^, ^30^, ^31^, ^32^, ^33^, ^34^] Therefore, herein, we used XAS to evaluate the formation of multinuclear Ag_*n*_S_*n*_ clusters in Cp‐1 ZFs and to gain direct insight into their structure. Furthermore, we used electronic spectroscopy techniques and gel electrophoresis to study the impact of Ag^I^ ions on the DNA complex of 1MEY# ZF protein (ZFP) (Figure 1C).

## Results and Discussion

### Qualitative Observation of XAS Spectra

**Figure 2:**
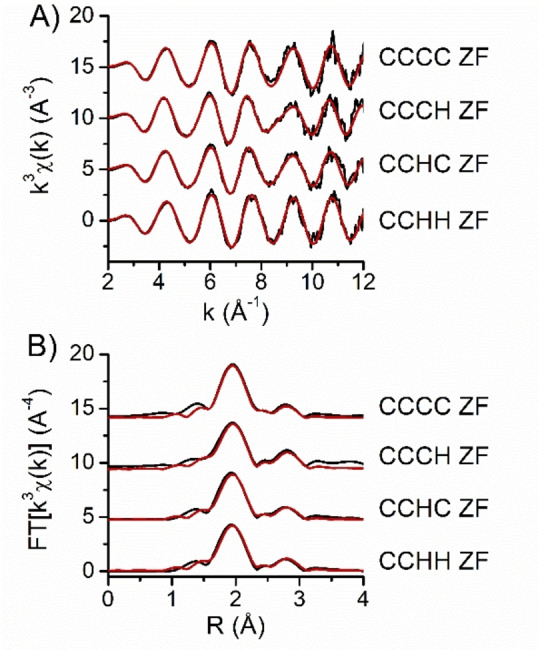
Experimental XAS data. A) K‐edge EXAFS spectra of silver fingers (black) together with the relative best‐fitting curves (red) obtained in the real space, then back‐transformed into the reciprocal space. B) Fourier‐transformed experimental EXAFS spectra (black) and the relative best‐fitting curves (red) obtained from least‐squares minimization of spectra generated with ab initio calculations.

The silver K‐edge XAS spectra were collected from ZF samples saturated with Ag^I^ ions (Ag^I^−ZFs), thus containing 2 molar equivalents of Ag^I^ for the CCHH ZF, 3 equivalents for CCHC and CCCH ZFs, and 4 equivalents for the CCCC ZF (see: Materials and Methods). These metal to ZF stoichiometric ratios were established by CD, as reported previously.^[27]^ The XANES spectra of Ag^I^−ZFs and the reference Ag^I^ glutathione complex (Ag^I^−GSH) are reported in Figure S1. The XANES region shows nearly identical spectral features for all examined Ag^I^−ZFs, suggesting only small variations in the Ag^I^ coordination environments among them. The k^3^‐weighted EXAFS spectra of Ag^I^−ZF complexes are shown in Figure 2A, along with the corresponding Fourier transform (FT) in Figure 2B. The best fitting curves are reported in red over the corresponding experimental spectra (black curves), both in the reciprocal space (Figure 2A) and in the real space (Figure 2B). All FT spectra show two main peaks centered at 2.0 Å and 2.8 Å, probably originating from single‐scattering contributions of two atomic shells. In all studied Ag^I^−ZF samples, the first‐shell peak is narrow and centered at around 2.0 Å, closely resembling the one observed in the Ag^I^−GSH reference complex featuring a digonal AgS_2_ coordination (Figure S2). Furthermore, the peak observed at about 2.8 Å in the FT spectra might indicate the presence of the second shell of Ag^I^ atoms forming a non‐bonding Ag⋅⋅⋅Ag interactions with the absorber, as previously described in the Ag^I^−GSH complex.^[35]^ A small contribution centered at 1.4 Å (Figure 2B) also present in all spectra might be due either to the presence of N or O ligands in the Ag^I^ coordination sphere, or to the window used to Fourier‐transform the spectra. The ab initio analysis of the spectra was necessary to define the number and nature of the Ag^I^ ligands, and the interatomic distances.

### Ab Initio EXAFS Analysis

In order to determine the Ag^I^−S coordination geometries, the FT spectra were fitted with a simple model including a first shell of S and N or O nearest neighbors, and a second shell of Ag atoms around the absorber. The best‐fitting parameters are reported in Table 1; the FT spectra for the best‐fitting curves together with the corresponding experimental data are shown in Figure 2B. As a template, we measured the spectrum of an Ag^I^−GSH complex (Figure S2B) formed in solution with 1 equivalent Ag^I^, corresponding to 1 Ag^I^ per Cys residue. In these conditions, Ag^I^−GSH forms mononuclear digonal AgS_2_ species, as proved by XRD and XAS studies.[^35^, ^36^, ^37^] Therefore, we measured empirically the amplitude reduction spectrum, by fixing the coordination number to two and allowing S_0_^2^ to vary. The estimated value of 0.79±0.04 was then fixed over the analysis of all Ag^I^−ZF samples, in which the coordination numbers were allowed to vary. The best‐fitting distances and DW factors of Ag^I^−GSH (Table 1) were used as starting values for all Ag^I^−ZF samples. The fits were performed in the real space, initially in the R range 1.0 to 2.3 Å, including only first‐shell Ag−S and Ag−N or Ag−O scattering contributions. The number of S (n_S_) and N/O (n_N/O_) atoms were allowed to vary: n_N/O_ was always estimated as 0 within the error, allowing us to exclude the presence of His nitrogen atoms or water molecules as Ag^I^ donors. The fitting range was then adjusted to [1.4–3.1] Å, in order to include the two main atomic shells around the absorber. The number of S donors and Ag−S distances (Table 1) are consistent with those reported in literature for digonal AgS_2_ sites in inorganic and biological Ag^I^ complexes.[^31^, ^32^, ^33^, ^36^, ^37^] For the CCCH Ag^I^−ZF, however, the number of sulfur atoms was estimated as 2.4±0.2 at 2.442±0.004 Å. A recent survey reported an average Ag−S distances of 2.39±0.03 Å in AgS_2_ thiolate sites, and of 2.51±0.05 Å in AgS_3_ sites.^[36]^ Based on that, the average Ag−S distance found in CCCH ZF cannot be unequivocally assigned to either AgS_2_ or AgS_3_ coordination. Instead, this distance is in very good agreement with that observed for mixed AgS_2_/AgS_3_ sites in metallothionein.^[34]^ For all complexes, we observed less than 1 Ag atom at ≈2.99 Å from the absorber (Table 1). The agreement between experimental and theoretical curves is excellent, as confirmed by low goodness‐of‐fit indexes (R_fit_ factor) reported in Table 1, both in the real space (Figure 2B) and in the reciprocal space (Figure 2A). The estimated Ag−Ag distances indicate the formation of non‐bonding Ag⋅⋅⋅Ag interactions similar to those found in Ag(HPen)⋅H_2_O.^[37]^

**Table 1:** Best‐fit parameters obtained from the analysis of the EXAFS spectra of Ag^I^−GSH and Ag^I^−ZF complexes.

	Ag−S			Ag−Ag			Fit parameters	
	*n* ^[b]^	*R* [Å]^[c]^	*σ* ^2^ [×10^−3^ Å^2^]^[d]^	*n* ^[b]^	*R* [Å]^[c]^	*σ* ^2^ [×10^−3^ Å^2^]^[d]^	Δ*E*_0_ [eV]^[e]^	*R*_fit_ [%]^[f]^
GSH	2.0^[a]^	2.408(5)	3.1(7)	0.8(4)	2.98(2)	9(5)	2.5(6)	0.5
CCCC ZF	2.0(1)	2.416(3)	3.5(3)	0.7(2)	2.99(1)	8(1)	3.8(3)	0.3
CCHH ZF	2.0(1)	2.404(4)	3.0(3)	0.3(2)	2.99(3)	6(3)	3.2(4)	0.4
CCHC ZF	2.0(3)	2.424(6)	4.8(5)	0.7(3)	2.98(2)	7(2)	3.5(5)	1.0
CCCH ZF	2.4(2)	2.443(4)	6.3(4)	0.9(3)	3.00(1)	6(1)	3.1(4)	0.5

[a] This value was fixed in the fit according to previous XRD and XAS characterizations.[^35^, ^36^, ^37^] [b] Number of atoms around the absorber. [c] Best‐fitting distances. [d] Dynamical parameters (Debye–Waller factors). [e]  Shift in the origin of the energy scale. [f]  Goodness‐of‐fit parameter.

**Figure 3:**
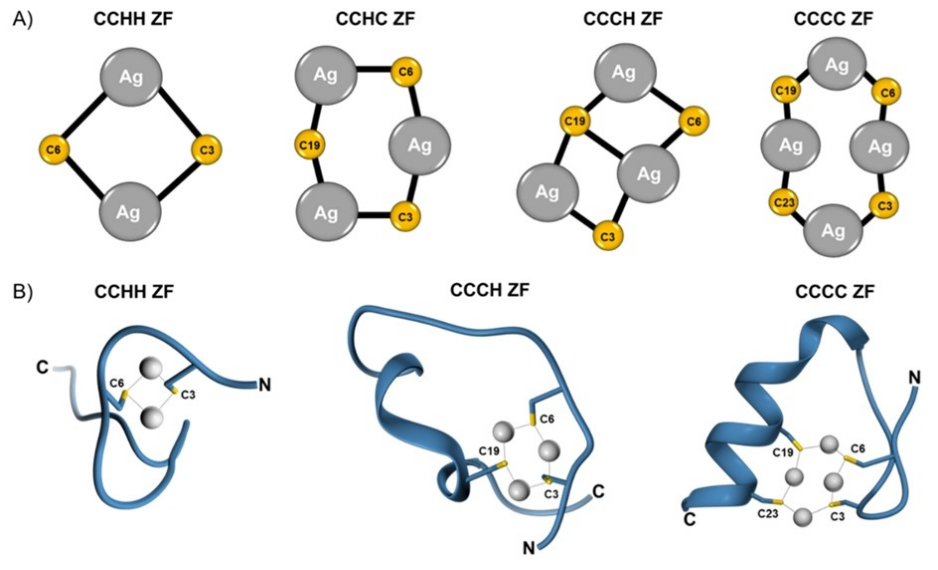
Structural features of Ag^I^−ZFs. A) Proposed structure of Ag_n_S_n_ ZF clusters probed in this study by EXAFS analysis. Each cysteine residue is numbered in accordance with ZF sequences from Figure 1B. B) Model structures of Ag_n_S_n_ ZF clusters obtained by QM/MM MD‐based simulations.^[27]^ C and N denote the C‐ and N‐terminal end of ZFs, respectively.

Overall, a simple two‐shells single‐scattering model allowed for a very good reproduction of the experimental signals, as confirmed also by the observation of the fitting curves back‐transformed and overlapped to the experimental data in the reciprocal space (Figure 2B). These XAS data prompted us to propose the structures of Ag_*n*_S_*n*_ clusters in Ag^I^−ZFs (Figure 3A), generally consistent with our previously reported models obtained by quantum mechanics/molecular mechanics (QM/MM) and molecular dynamics simulations (presented in Figure 3B for a comparison).^[27]^ The average Ag−S bond length of the CCCH Ag^I^−ZF is longer than in other Ag^I^−ZFs. As a result, the Ag^I^−thiolate cluster in this particular ZF tends to form a more twisted‐chain structure, probably inducing minor changes in the secondary structure as a whole (also indicated by CD, see below).

### Relationship between the Ag^I^ Coordination Environment and Structures of Ag^I^−ZFs CCCH and CCHC.

**Figure 4:**
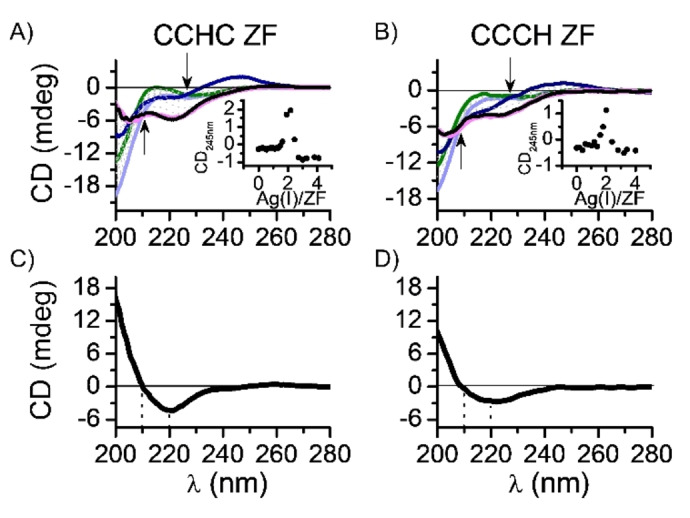
CD titrations of 25 μM metal‐free A) CCHC ZF (examined in this study) and B) CCCH ZF (data obtained previously)^[27]^ in 20 mM TES buffer (pH 7.0) with AgNO_3_ solution. The light blue, green, dark blue, magenta, and black lines indicate 0, 1, 2, 3, and 4 equivalents of added Ag^I^, respectively. Arrows indicate observable isodichroic points. The insets represent the CD signal at 245 nm as a function of the Ag^I^‐to‐ZF molar ratio. C) and D) the differential spectra obtained by subtraction of metal‐free CCHC and CCCH ZF CD spectrum from fully Ag^I^‐loaded CCHC and CCCH ZF spectrum, respectively. The dotted lines represent ellipticity changes at 210 and 220 nm.

Because EXAFS data revealed differences in Ag^I^ coordination geometry between CCCH ZF and CCHC ZF, we decided to monitor the Ag^I^ binding to CCHC ZF (which was not studied before) using CD spectroscopy, to see if differences in Ag^I^−thiolate cluster geometries in the respective Ag^I^−ZFs could affect the folding process. The 0 to 4 equivalents of Ag^I^ were titrated to the CCHC ZF. The resulting CD spectra (Figure 4) featured various negative and positive bands in the 200–250 nm range indicating the formation of multiple Ag^I^ complexes. As shown in Figure 4A, the CD band at 245 nm (+, weak) occurred around 2 equivalents of Ag^I^; and was replaced between 2 and 3 equivalents by bands at 205 nm (−, moderate) and 220 nm (−, moderate). No spectral changes occurred above 3 equivalents of Ag^I^. The signal at 245 nm originates from S→Ag^I^ LMCT, while signals at 200–230 nm are mostly due to secondary structure features. Similarly, the band at 245 nm was previously reported for the CCHH, CCCH and CCCC ZFs.^[27]^ Its maximum intensity was observed at exactly 2 equivalents of Ag^I^ for all studied ZFs, and hence was assigned to the Ag_2_S_2_ cluster formation. Such clusters may be formed in several ways, including interpeptide complexes. Nevertheless, the results presented here are consistent with a transition from the Ag_2_S_2_ cluster to the Ag_3_S_3_ cluster between 2 and 3 equivalents of Ag^I^ during titration of CCHC ZF with a parallel secondary structure alteration.^[38]^ An isodichroic point at 227 nm present at 2 mol equivalents of Ag^I^ is blue shifted to 210 nm for 3 mol equivalents of Ag^I^ (Figure 4, black arrows), indicative of a β‐structure‐coil transition.^[39]^ Therefore, the movement of the isodichroic point along the formation of Ag^I^−thiolate clusters can be related to a subsequent secondary structure transition. Similarly to the previously studied CCCH Ag^I^−ZF, the maximal number of Ag^I^ ions bound to CCHC ZF was equal to the number of Cys residues, yielding a Ag_3_L stoichiometry. Interestingly, the CD spectra obtained herein for the CCHC Ag^I^−ZF differed slightly from those reported previously for the CCCH Ag^I^‐ZF at 3 mol equivalents of Ag^I^ (Figure 4B), indicating a secondary structure difference.^[27]^ To further probe the CD bands at 200–260 nm in the saturated Ag^I^ complex, the differential spectra of the tested peptides were included in Figure 4C and 4D. For that purpose the metal‐free ZF spectrum was subtracted from the Ag^I^‐saturated ZF peptide spectrum. Because metal‐free ZF spectrum adopts a random coil structure, the contribution between 200–230 nm derived from the intrinsic chirality of the component amino acids was subtracted. Such differential CD spectrum is solely derived from the Ag^I^‐bound peptide chain, containing contributions from the peptide fold (secondary structure) and thiolate (or imidazole) to Ag^I^ LMCT bands. Therefore, the differential spectra are not the clear‐cut indicators of secondary structure formation. Nevertheless, taking into account conserved sequences of both peptides and the same number of Cys and His ligands, we can conclude that the differences between these spectra reflect secondary structure differences. The AgS_2_ site in CCHC ZF induced the formation of a positive signal at 210 nm and a negative one at 220 nm, corresponding to a combination of random coil and β‐elements (Figure 4C). Its differential spectrum resembles those obtained elsewhere for CCHH Ag^I^−ZF (Figure S3A).^[27]^ The mixed AgS_2_/AgS_3_ site in CCCH ZF induced the partial formation of α‐ and β‐elements, as proved by negative signals at 210 nm and 220 nm (Figure 4D), similar to those observed for the CCCC Ag^I^−ZF cluster with a ββα fold (Figure S3B).^[27]^ This shows that relationship between Cys residues positions and the arrangement of the silver coordination site will contribute to secondary structural elements.

### Impact of Ag^I^ Ions on ZF‐DNA Interaction

**Figure 5:**
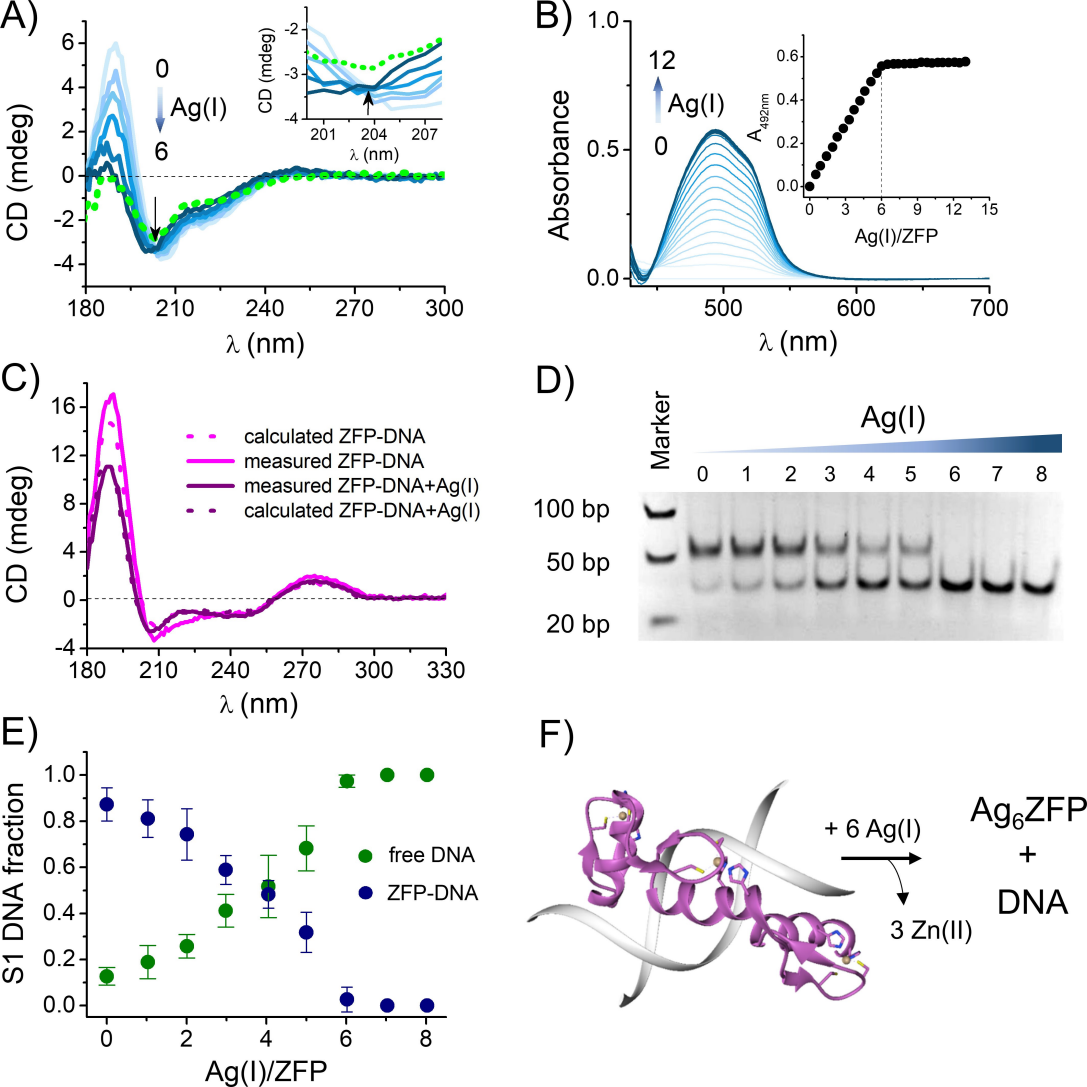
Ag^I^ binding to CCHH 1MEY# ZFP and damage of ZF structure and DNA‐ZFP complex. A) CD titration of 16 μM Zn^II^‐loaded 1MEY# ZFP with Ag^I^ in 10 mM HEPES buffer pH 7.4. The arrow represents the increasing equivalents of added Ag^I^. The dotted green line represents the CD spectrum of 1MEY# ZFP at a 5‐fold excess of EDTA. The isodichroic point zoomed in the inset is marked with a black arrow. B) Zn^II^ transfer from 2.5 μM 1MEY# ZFP to 89 μM PAR during a titration with 0–12 equivalents of Ag^I^. The measurement was performed in 50 mM HEPES buffer (pH 7.4). Inset indicates that six Ag^I^ ions are bound to 1MEY# ZFP (two Ag^I^ per each ZF motif) and three Zn^II^ are dissociated. C) Comparison of the CD spectra obtained for the equimolar (12–12 μM) mixtures of Zn^II^‐saturated 1MEY# ZFP and specific S1 DNA in the presence and absence of 6 equivalents of Ag^I^ in 10 mM HEPES, 90 mM NaClO_4_ buffer (pH 7.4). The dotted lines represent the CD spectra calculated by summing the appropriate protein and DNA component spectra. The applied 6 equivalents of Ag^I^ (2 equiv of Ag^I^ per each ZF unit) destroyed the 1MEY# ZFP structure. D) Electrophoretic gel mobility shift assay of 1MEY# ZFP with the specific S1 DNA probe in the presence of increasing equivalents of Ag^I^ indicates that 6 equivalents of Ag^I^ per 1MEY# ZFP (2 equiv per each ZF unit) results in a complete dissociation of 1MEY# ZFP‐DNA complex. A 0.25 eq. protein excess was applied over S1 DNA in 10 mM HEPES, 150 mM NaClO_4_ buffer (pH 7.4). E) Distribution of S1 DNA along with the Ag^I^ titration of ZFP‐DNA complex. The fractions of ZFP‐bound and free DNA were calculated based on the intensities of five independent electrophoretic gel mobility shift assays. Band intensity calculations were performed by ImageJ.^[43]^ Deviation of Ag^I^/ZFP ratios from experiment to experiment varied by less than 2 %. Therefore, these error bars are not presented as they would be fully contained within the symbols. The same regards the DNA fractions for Ag^I^/ZFP ratios of 7 and 8. F) Schematic representation of consequences of Ag^I^ binding to 1MEY ZFP complexed with DNA (PDB: 1MEY).

In order to look deeper into the toxicological consequences of the formation of Ag^I^−thiolate clusters in a ZFP, we performed a CD‐based Ag^I^ titration of the Zn^II^‐saturated 1MEY# ZFP[^40^, ^41^] (*holo*‐protein consisting of three finger domains) and its DNA complex. The 1MEY# ZF *holo*‐protein exhibited characteristic negative bands at 205 nm and 225 nm (Figure 5A), indicating the ββα structure formation. This well‐defined structure of the CCHH zinc fingers in the *holo*‐protein collapsed gradually upon the addition of Ag^I^ ions as indicated by the CD spectra presented in Figure 5A. The finger units seem to behave independently, as suggested by the isodichroic point at 204 nm. The CD spectrum changed by increasing the Ag^I^ excess up to ≈2 Ag^I^ equivalents per ZF (up to ≈6 equiv Ag^I^ per protein). The final CD spectrum of the Ag^I^‐saturated 1MEY# ZFP (Figure 5A) is very similar to the spectrum recorded for 1MEY# ZFP demetalated by the EDTA excess (green dotted line, Figure 5A). To confirm the Zn^II^ release from 1MEY# ZFP by the Ag^I^ binding, the chromophoric Zn^II^ chelating probe 4‐(2‐pyridylazo)resorcinol (PAR) was added to the *holo*‐protein, followed by stepwise addition of Ag^I^ ions, up to 12 per protein. Monitoring the Zn^II^ dissociation from 1MEY# ZFP is possible due to the formation of a ZnH_*x*_(PAR)_2_ complex intensely absorbing light at 492 nm (*ϵ*=71 500 M^−1^ cm^−1^).^[42]^ The Ag^I^ complex of PAR does not absorb at 492 nm, as reported,^[27]^ thus enabling a direct Zn^II^ dissociation quantitation. Titrations of 1MEY# ZF *holo*‐protein with Ag^I^ in the presence of a PAR excess yielded a clear endpoint at ca. 6 equivalents of Ag^I^, based on four independent titrations (Figure 5B), in perfect accordance with an expectation of complete Zn^II^ displacement from CCHH ZFs by the formation of Ag_2_S_2_ clusters (Figure 3A). Together with the CD titration data, this result indicates that Zn^II^ ions can be completely displaced from ZFPs during Ag^I^ overload, accompanied by a loss of three‐dimensional ZFP ββα structure. To further explore possible biological implications of this finding, the DNA binding of 1MEY# ZF *holo*‐protein in the presence of Ag^I^ was also studied. CD spectroscopy revealed that the addition of cognate S1 DNA to the *holo*‐protein enhanced the intensity of CD bands as compared to the curve calculated by summing up the component spectra of 1MEY# ZFP and S1 DNA in the appropriate ratio (Figure 5C). This indicated the formation of a tight ZFP‐DNA complex. Nevertheless, the addition of ≈6 equivalents of Ag^I^ per protein resulted in a significant decrease of the CD spectral intensity, down to the calculated value assuming no protein DNA interaction (Figure 5C) suggesting that the Ag^I^ ions could efficiently compete for 1 MEY#ZFP with DNA. This result was further supported by an electrophoretic mobility shift assay (EMSA) in a native polyacrylamide gel. Figure 5D shows how in the Ag^I^ absence, the 1MEY# ZFP binds its cognate S1 DNA, increasing its apparent size from the original 34 bp to ca. 50 bp. Upon the Ag^I^ addition, the expected gradual DNA release occurred. At 6 Ag^I^ equivalents (2 equiv per ZF motif), the gel mobility shift vanished, and only the original DNA band was visible in the gel at 34 bp. As demonstrated in Figure 5E, the sigmoidal pattern of the of ZFP‐free DNA band intensity increase with Ag^I^ suggests a cooperative mechanism of Ag^I^ binding to ZFPs. Hypothetically, the replacement of one Zn^II^ ion with the Ag_2_S_2_ cluster destabilizes the ZFP‐DNA interaction, facilitating the Ag^I^ assault on the other two ZFs. These experiments indicate a strong ability of Ag^I^ ions to prevent the interaction of the CCHH type ZFPs with DNA at a 2 : 1 Ag^I^ to ZF motif ratio under the applied conditions.

The results presented above demonstrated that all major ZF types can be targeted by Ag^I^ ions in physiological conditions, which results in the loss of their native three‐dimensional structure. The replacement of Zn^II^ in ZFs by Ag^I^‐thiolate clusters described in detail in this study will thus disable the physiological functions of ZFPs, which are based on molecular recognition empowered by native ZF conformations. This was demonstrated directly here, by observing the Ag^I^‐dependent detachment of 1MEY# ZFP, bearing three CCHH ZF domains, from its cognate DNA (Figure 5D–F). Our findings indicate that Ag^I^ ions, including those released intracellularly from AgNPs may interfere with a manifold of cellular processes involved in gene expression, genomic integrity, cell growth, differentiation, and DNA repair.

## Conclusion

The knowledge regarding Zn^II^ substitution in proteins by other metal ions is critical for the assessment of possible toxicological consequences of such chemical reactions. The Zn^II^ replacement by another metal ion may lead to the formation of stronger metal complexes with disturbed coordination geometry and/or unwanted chemical reactivity. Silver nanoparticles (AgNPs) are widely present in the human environment, enabling Ag^0^ and Ag^I^ bioavailability. Once in the cell cytosol, they end up as Ag^I^−thiolate complexes suggesting that cysteine‐rich proteins and peptides are their preferred biological targets. Herein, we characterized in detail the structures of Ag^I^ complexes in sequentially diverse ZFs, highlighting a formation of Ag_*n*_S_*n*_ clusters (*n*=2, 3 or 4) with the predominant AgS_2_ binding mode. The average Ag−S bond lengths for CCHH, CCHC, and CCCC ZFs are in good agreement with average Ag−S bond lengths for Ag−thiolate clusters reported previously. We also showed that the loss of native ZFP chain conformation caused by Zn^II^ replacement with Ag^I^ directly destroyed the specific DNA binding by a three ZF array 1MEY# ZFP. In conclusion, our findings provide strong chemical evidence for the genotoxic potential of silver and lay a solid foundation for further research on silver toxicity.

## Conflict of interest

The authors declare no conflict of interest.

1

## Supporting information

As a service to our authors and readers, this journal provides supporting information supplied by the authors. Such materials are peer reviewed and may be re‐organized for online delivery, but are not copy‐edited or typeset. Technical support issues arising from supporting information (other than missing files) should be addressed to the authors.

Supporting Information

## Data Availability

The data that support the findings of this study are available from the corresponding author upon reasonable request.
