# Supplementary material for: Structures of Silver Fingers and a Pathway to Their Genotoxicity
Source: Angew Chem Int Ed Engl. 2022 Feb 3;61(12):e202116621. doi: 10.1002/anie.202116621 (PMC9303758; doi:10.1002/anie.202116621)
Supplement: Supplementary file 1 — Supporting Information [file ANIE-61-0-s001.pdf]

Angewandte Chemie
Eine Zeitschrift der Gesellschaft Deutscher Chemiker

Supporting Information

Structures of Silver Fingers and a Pathway to Their Genotoxicity

K. Kluska, G. Veronesi, A. Deniaud, B. Hajdu, B. Gyurcsik, W. Bal, A. Krężel**

Supporting Information
©Wiley-VCH
69451 Weinheim, Germany

Structures of Silver Fingers and a Pathway to Their Genotoxicity

Katarzyna Kluska[a], Giulia Veronesi[b], Aurélien Deniaud[b], Bálint Hajdu[c], Béla Gyurcsik[c], Wojciech Bal*[d], and Artur Krężel*[a]

[a] K. Kluska, Prof. A. Krężel
Department of Chemical Biology, Faculty of Biotechnology
University of Wrocław
F. Joliot-Curie 14a, 50-383, Wrocław
E-mail: artur.krezel@uwr.edu.pl

[b] Dr. G. Veronesi, Dr. A. Deniaud
Univ. Grenoble Alpes, CNRS, CEA, IRIG, Laboratoire de Chimie et Biologie des Métaux,
F-38000 Grenoble, France

[c] B. Hajdu, Prof. B. Gyurcsik
Department of Inorganic and Analytical Chemistry, Faculty of Science and Informatics
University of Szeged
H-6720 Szeged, Dóm tér 7, Hungary

[d] Prof. W. Bal
Institute of Biochemistry and Biophysics
Polish Academy of Sciences
Pawińskiego 5a, 02-106 Warsaw, Poland
E-mail: wbal@ibb.waw.pl

Abstract: Recently, we demonstrated that Ag(I) can directly replace Zn(II) in zinc fingers (ZFs). The cooperative binding of Ag(I) to ZFs leads to a thermodynamically irreversible formation of silver clusters destroying the native ZF structure. Thus, a reported loss of biological function of ZF proteins is a likely consequence of such replacement. Here, we report the X-ray absorption spectroscopy (XAS) study of Ag_nS_n clusters formed in ZFs to probe their structural features. Selective probing of local environment around Ag(I) by XAS showed the predominance of digonal Ag(I) coordination to two sulfur donors, coordinated with an average Ag−S distance at 2.41 Å. No Ag−N bonds were present. A mixed AgS_2/AgS_3 geometry was found solely in the CCCH Ag(I)-ZF. We also show that cooperative replacement of Zn(II) ions with the studied Ag_2S_2 clusters occurred in a three-ZF transcription factor protein 1MEY#, leading to a dissociation of 1MEY# from the complex with its cognate DNA.

Table of Content

Experimental Procedures 3
a. Materials 3
b. Peptide synthesis and purification 3
c. Expression of Cp-1-like modified version of 1MEY ZFP 3
d. EXAFS spectroscopy 3
e. EXAFS data analysis 3
f. Circular Dichroism (CD) 4
g. Spectrophotometric monitoring of Zn(II) release from 1MEY# ZFP 4
h. Electrophoretic mobility shift assay (EMSA) 4
Supporting Figures 5
References 7

Experimental Procedures

a. Materials

$AgNO_3$, $ZnSO_4{\cdot}7H_2O$, 1,2-ethanedithiol (EDT), thioanisole, anisole, triisopropylsilane (TIPS), 2-[(2-hydroxy-1,1-bis(hydroxymethyl)ethyl)amino]ethanesulfonic acid (TES) were purchased from Sigma-Aldrich (Merck). The 5,5′-dithiobis(2-nitrobenzoic acid) (DTNB) was purchased from TCI America. The metal-chelating resin Chelex100® was from BioRad. The *N*,*N*-dimethylformamide (DMF), HCl were from VWR Chemicals. Acetonitrile (ACN) was from Merck Millipore. Acetic anhydride, diethyl ether, dichloromethane (DCM) were from Avantor Performance Materials Poland (Gliwice, Poland). Tris(2-carboxyethyl)phosphine hydrochloride (TCEP), 1-methyl-2-pyrrolidinone (NMP), *N*,*N*,*N*,*N*-tetramethyl-*O*-(1*H* benzotriazol-1-yl)uranium hexafluorophosphate (HBTU), trifluoroacetic acid (TFA), N,N-diisopropylethylamine (DIEA), piperidine, TentaGel S Ram and Fmoc-protected amino acids were obtained from Iris Biotech GmbH (Marktredwitz, Germany). The concentrations of peptides were determined spectrophotometrically using DTNB as previously described.[1] The concentration of stock solutions of metal ion salts were 0.05 M, confirmed by representative series of ICP-MS measurements. To eliminate trace metal ion contamination all pH buffers were treated with Chelex 100® resin and degassed over 2 h prior use.

b. Peptide synthesis and purification

All investigated zinc finger peptides were synthesized via solid phase synthesis on TentaGel S Ram resin (substitution 0.22 mmol/g) using Fmoc-strategy and Liberty 1 microwave-assisted synthesizer (CEM). The reagent excess, cleavage and purification were performed as previously described.[2,3] Acetic anhydride was used for N-terminal acetylation, then peptides were cleaved from the resin with a mixture of TFA/anisole/thioanisole/EDT/TIPS (88/2/3/5/2 v/v/v/v/v) over a period of 2.5 h followed by precipitation in cold (-70°C) diethyl ether. The crude peptides were collected by centrifugation, dried and purified using HPLC (Dionex Ultimate) on Phenomenex C18 columns using a gradient of ACN in 0.1% TFA/water from 0% to 40% over 20 min. The purified peptides were identified by Bruker compact Q-TOF ESI mass spectrometer (Bruker Daltonics). The identified and calculated monoisotopic masses are listed in Table S1.

c. Expression of Cp-1-like modified version of 1MEY ZFP

To investigate the interactions between a zinc finger (ZF) protein with Ag(I), a construct of modified version of the 1MEY ZFP (later referred to as 1MEY#) which contains three Cp-1-like CCHH zinc finger motifs was designed as previously described.[4] Then, the purified and verified plasmid was transformed into BL21 (DE3) *E. coli* cells. Bacteria were cultured in a rich full growth medium until OD at 600 nm reached 0.3, when the protein expression was induced by 0.2 mM IPTG at 20°C for 18 hours. Cells were collected and resuspended in a buffer containing 500 mM NaCl, 100 mM HEPES (pH 8.2), and 10 mM imidazole, and then sonicated. The expressed protein was subjected to Ni(II)-affinity chromatography as previously described.[4]

d. EXAFS spectroscopy

Prior to EXAFS analysis, the samples of ZFs were dissolved in 50 µl of 20 mM TES pH 7.0 and saturated with Ag(I) at molar excess 4 (CCCC ZF), 3 (CCCH and CCHC ZFs) or 2 (CCHH ZF). Then the samples were freeze-dried and stored at -80°C until the XANES/EXAFS experiments. Just before the experiment, the samples were resuspended with milliQ water and a 50 µl drop of each solution was deposited on a sample holder equipped with kapton windows and immediately frozen in liquid N_2. The Ag K-edge XAFS experiments were carried out at the beamline CRG-FAME-BM30 at the European Synchrotron Radiation Facility (ESRF, France).[5] The Ag K absorption edge was scanned in the energy range 25.300-26.480 keV with a nitrogen-cooled Si(220) double-crystal monochromator.[6] The incoming photon energy was calibrated with an Ag metallic foil, by defining the first inflexion point of its XAFS spectrum at 25.514 keV. The experiment was performed at ~15 K in a liquid He cryostat. The spectra were recorded in fluorescence mode, with a 30-element Ge solid state detector (Canberra). The number of scans per sample was chosen to obtain ~10^6 total counts above the absorption edge. It resulted in four to five spectra per sample that were collected and averaged.

e. EXAFS data analysis

XAS data reduction and normalization was performed with standard methods using the Demeter suite.[7] The EXAFS spectra were extracted from the raw data and analyzed using the Athena and Artemis softwares, respectively.[7] Theoretical scattering amplitudes and phase shifts were calculated by means of the ab initio code FEFF6 implemented in Artemis.[8] The amplitude reduction factor S_0^2 was measured empirically over a silver-glutathione 1:1 reference compound: the estimated value of 0.79 ± 0.04 was fixed during the

analysis. The EXAFS spectra were Fourier-transformed over the [2.2; 12] $Å^{-1}$ k-range, then fitted in the R space in the range [1; 3.1] Å with multiple k-weights, using a modified Levenberg−Marquardt method as the minimization algorithm. A shift in the threshold energy (ΔE_0) was always set as a global variable.

f. Circular Dichroism (CD)

CD spectra of Ag(I) binding to metal-free CCHC ZF peptide was recorded on a J-1100 Jasco spectropolarimeter at 25°C in a 2 mm quartz cuvette under constant nitrogen flow with a 100 nm/min scan speed over the range of 200 – 260 nm and 200 – 400 nm, respectively. Final spectra were averaged from 3 independent scans. The spectroscopic titration of 25 µM ZF peptide with Ag(I) was performed in degassed and chelexed 20 mM TES buffer (pH 7.0) without TCEP (to avoid AgCl precipitation from the Cl- counterion of TCEP), respectively. All samples were equilibrated over 2 min after the addition of each portion of 7 mM $AgNO_3$ solution.

CD spectra to monitor Ag(I) binding to Zn(II)-saturated 1MEY# ZFP-DNA complex were recorded on a J-1500 Jasco spectropolarimeter at 25°C under the constant nitrogen flow with a 20 nm/min scan speed over the range of 180 – 330 nm, with 1 nm resolution and 2 s response time. Synchrotron radiaton (SR) CD spectra were recorded at the CD1 beamline of the storage ring ASTRID at the Institute for Storage Ring Facilities (ISA), University of Aarhus, Denmark.[9,10] All spectra were recorded with 1 nm steps and a dwell time of 2 s per step, using l = 0.1 or 0.2 mm quartz cells (SUPRA-SIL, Hellma GmbH, Germany), in the wavelength range of 170–330 nm. Each sample containing 12-20 µM protein and increasing amounts of $AgClO_4$ in the presence or absence of S1 DNA (identical to that used in the electrophoretic mobility shift assays) was prepared separately and incubated at room temperature for 5 minutes prior measurement in 10 mM HEPES buffer (pH 7.4). The ionic strength was adjusted to 0–120 mM with $NaClO_4$

g. Spectrophotometric monitoring of Zn(II) release from 1MEY# ZFP

The transfer of Zn(II) from 1MEY# ZFP to PAR during Ag(I) titration was performed spectrophotometrically using an Evolution 220 spectrophotometer (Thermo Scientific) in a 1 cm quartz cuvette, with the spectra recorded between 700 and 220 nm. 450 µl samples containing 1-3 µM protein and 89 µM PAR were titrated in 50 mM HEPES buffer pH 7.4 with 5 µl aliquots of $AgClO_4$. Special care was taken to avoid the adhesion of $Zn(PAR)_2$ complex, which can happen on plastic surfaces. The samples were incubated at 25°C for 5 minutes after each injection, which proved to be sufficient to reach equilibrium. The exact concentration of $Zn(PAR)_2$ complex was calculated based on absorbances at 492 nm using the effective molar absorbance coefficient at pH 7.4 (71,500 M^{-1} cm^{-1}).[11] At the titration endpoint the spectrophotometrically determined concentration of released Zn(II) was consistent with the values calculated from the zinc finger *holo*-protein concentration.

h. Electrophoretic mobility shift assay (EMSA)

The 34bp DNA (later referred to as S1 DNA) containing the forward 5’-GAATTCCTGCTGAGAGGCAGAAACATAGGGGTCG–3’ sequence which included the 1MEY# ZFP target sequence (underlined) and the reverse sequence 3’-CTTAAGGACGACTCTCCGTCTTTGTATCCCCAGC-5’, was obtained by the hybridization of the two oligonucleotides obtained by solid phase synthesis (Invitrogen) and was further used for EMSA experiments performed in 5 µl of a 10 mM HEPES buffer, pH 7.4, containing 10% (V/V) glycerol, 0–150 mM $NaClO_4$, 0.70 µM S1 DNA, 0.88 µM 1MEY# ZFP and $AgClO_4$ at concentrations resulting in 0:1 to 8:1 Ag(I):ZFP ratios. ZFP-DNA mixtures were incubated at room temperature for 15 minutes. This was followed by the addition of various equivalents of $AgClO_4$ and a further 5 minutes incubation prior gel loading. 5 µl FastRuler Ultra Low Range DNA Ladder (Thermo Scientific) was used as reference. The reaction mixtures were separated on a 6% (m/V) native polyacrylamide gel containing 12.5 mM Tris buffer and 96 mM glycine during vertical electrophoresis at 100 V, 30 min, 4°C (Mini-PROTEAN Tetra Cell). DNA probes were visualized by 0.5 µg/ml EtBr staining for 15 minutes. Five independent assays were performed.

Supporting Table and Figures

Table S1. List of experimental (M_{exp}) and calculated (M_{calc}) molecular masses of examined ZF peptides.

ZF	M_{exp} (g/mol)	M_{calc} (g/mol)
CCHH	2894.61	2894.34
CCHC	2860.81	2860.32
CCCH	2860.95	2860.32
CCCC	2826.06	2826.33

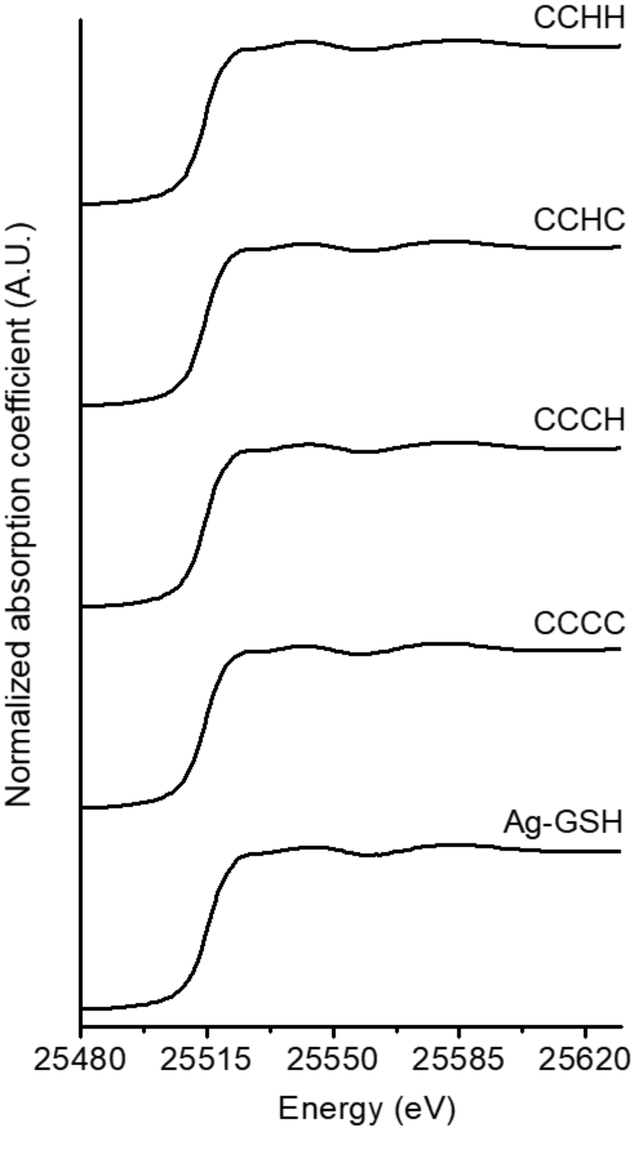

Figure S1 - Silver K-edge XANES spectra obtained for the examined Ag(I)-ZFs and model Ag(I)-GSH complex.

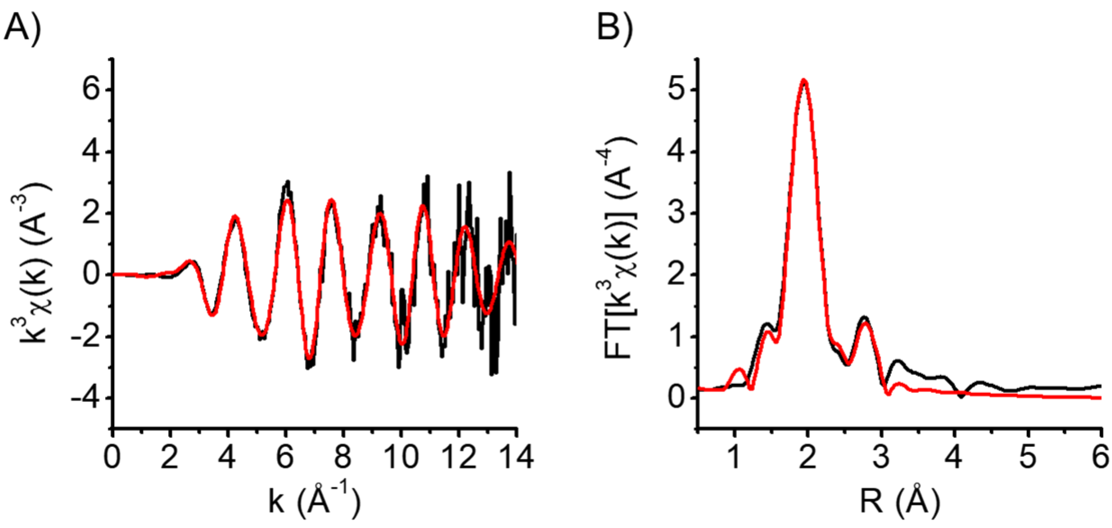

Figure S2 - Experimental XAS data obtained for the model Ag(I)-GSH complex. (A) Silver K-edge EXAFS spectrum of Ag(I)-GSH (black) together with the relative best-fitting curve (red). (B) Fourier-transformed experimental EXAFS spectrum (black) and the relative best-fitting curve (red) obtained from least-squares minimization of spectrum generated with *ab initio* calculations.

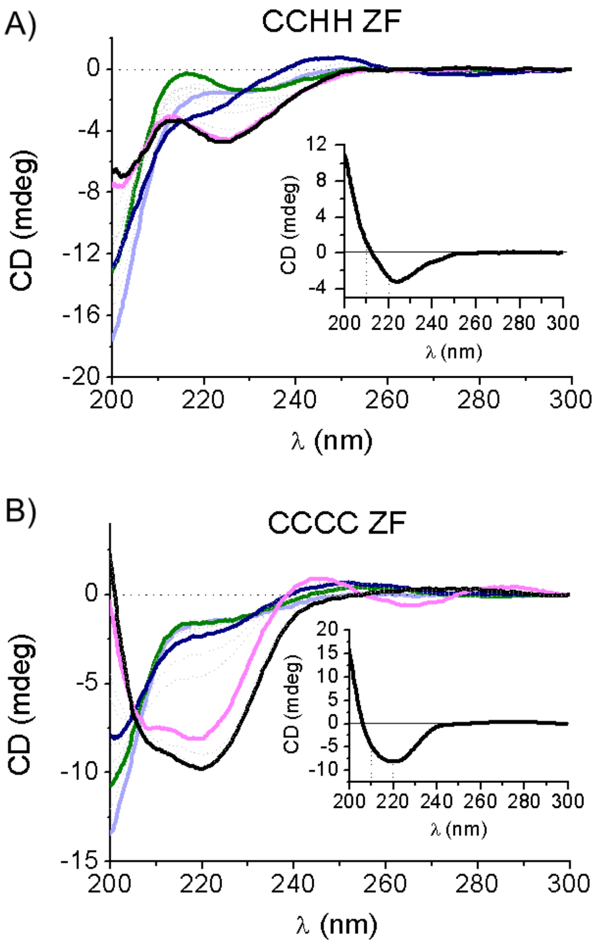

Figure S3 - Structural changes of silver fingers. CD titrations of 25 µM metal-free (A) CCHH ZF and (B) CCCC ZF (data derived from previously reported study)[12] in 20 mM TES buffer (pH 7.0) with $AgNO_3$ solution. The light blue, green, blue, magenta and black lines indicate 0, 1, 2, 3 and 4 equiv of added Ag(I). The insets represent differential spectra obtain by subtraction of apo-ZF CD spectrum from fully Ag(I)-loaded ZF spectrum.

References

[1] P. Eyer, F. Worek, F. Kiderlen, G. Sinko, A. Stuglin, V. Simeon-Rudolf, E. Reiner, *Anal. Biochem.* **2003**, *312*, 224–227
[2] A. Krężel, J. Wójcik, M. Maciejczyk, W. Bal, *Inorg. Chem*. **2011**, *50*, 72–85.
[3] K. Kluska, J. Adamczyk, A. Krężel, *Metallomics* **2018**, *10*, 248–263.
[4] A. Belczyk-Ciesielska, B. Csipak, B. Hajdu, A. Sparavier, M. N. Asaka, K. Nagata, B. Gyurcsik, W. Bal, *Metallomics* **2018**, *10*, 1089–1098.
[5] O. Proux, X. Biquard, E. Lahera, J. J. Menthonnex, A. Prat, O. Ulrich, Y. Soldo, P. Trévisson, G. Kapoujvan, G. Perroux, P. Taunier, D. Grand, P. Jeantet, M. Deleglise, J. P. Roux, J. L. Hazemann, *12th International Conference on XAFS, Physica Scripta* **2005**, *115*, 970–973.
[6] O. Proux, V. Nassif, A. Prat, O. Ulrich, E. Lahera, X. Biquard, J. J. Menthonnex, J. L. Hazemann, *J. Synchrotron Radiation* **2006**, *13*, 59–68.
[7] B. Ravel, M. Newville, *J. Synchrotron Radiation* **2005**, *12*, 537–541.
[8] S. I. Zabinsky, J. J. Rehr, A. Ankudinov, R. C. Albers, M. J. Eller, *Phys. Rev.* **1995**, *52*, 2995–3009.
[9] A. J. Miles, S. V. Hoffmann, Y. Tao, R. W. Janes, B. A. Wallace, *Spectroscopy* **2007**, *21*, 245–255.
[10] A. J. Miles, R. W. Janes, A. Brown, D. T. Clarke, J. C. Sutherland, Y. Tao, B. A. Wallace, S. V. Hoffmann, *Synchrotron Radiat.* **2008**, *15*, 420–422.
[11] A. Kocyła, A. Pomorski, A. Krężel, *J. Inorg. Biochem*. **2015**, *152*, 82–92.
[12] K. Kluska, M. D. Peris-Díaz, D. Płonka, A. Moysa, M. Dadlez, A. Deniud, W. Bal, A. Krężel, *Chem. Commun. (Camb.)* **2020**, *56*, 1329–1332.
